# Author Correction: Endogenous control of inflammation characterizes pregnant women with asymptomatic or paucisymptomatic SARS-CoV-2 infection

**DOI:** 10.1038/s41467-021-26014-3

**Published:** 2021-09-27

**Authors:** Sara De Biasi, Domenico Lo Tartaro, Lara Gibellini, Annamaria Paolini, Andrew Quong, Carlene Petes, Geneve Awong, Samuel Douglas, Dongxia Lin, Jordan Nieto, Francesco Maria Galassi, Rebecca Borella, Lucia Fidanza, Marco Mattioli, Chiara Leone, Isabella Neri, Marianna Meschiari, Luca Cicchetti, Anna Iannone, Tommaso Trenti, Mario Sarti, Massimo Girardis, Giovanni Guaraldi, Cristina Mussini, Fabio Facchinetti, Andrea Cossarizza

**Affiliations:** 1 Department of Medical and Surgical Sciences for Children and Adults, University of Modena and Reggio Emilia School of Medicine, Modena, Italy; 2 Fluidigm Corporation, South San Francisco, CA USA; 3 College of Humanities, Arts and Social Sciences, Flinders University, Bedford Park, SA 5042 Australia; 4 Infectious Diseases Clinics, AOU Policlinico and University of Modena and Reggio Emilia, Modena, Italy; 5 Labospace, Milano, Italy; 6 Department of Surgery, Medicine, Dentistry and Morphological Sciences, University of Modena and Reggio Emilia School of Medicine, Modena, Italy; 7 Department of Clinical Pathology, AOU Policlinico, Modena, Italy; 8 Department of Anesthesia and Intensive Care, AOU Policlinico and University of Modena and Reggio Emilia, Modena, Italy; 9 National Institute for Cardiovascular Research, Bologna, Italy

**Keywords:** Viral infection, Inflammation, Neutrophils, Translational immunology

Correction to: *Nature Communications* 10.1038/s41467-021-24940-w, published online 29 July 2021.

The original version of this Article contained an error in Table 1. The correct version of the first row of the 2nd, 3rd, 5th and 7th columns states ‘CTR’, ‘PN’, ‘CTR vs PN’ and ‘PN vs PP’, instead of the original, incorrect ‘HD’, ‘NP’, ‘CTR vs NP’ and ‘CTR vs PP’. This has been corrected in both the PDF and HTML versions of the Article.

