# Supplementary material for: Endogenous control of inflammation characterizes pregnant women with asymptomatic or paucisymptomatic SARS-CoV-2 infection
Source: Nat Commun. 2021 Jul 29;12:4677. doi: 10.1038/s41467-021-24940-w (PMC8322155; doi:10.1038/s41467-021-24940-w)
Supplement: Supplementary file 1 — Supplementary Information [file 41467_2021_24940_MOESM1_ESM.pdf]

Supplementary Figures

De Biasi et al.

Endogenous control of inflammation characterizes
pregnant women with asymptomatic or mild SARS-CoV-2 infection

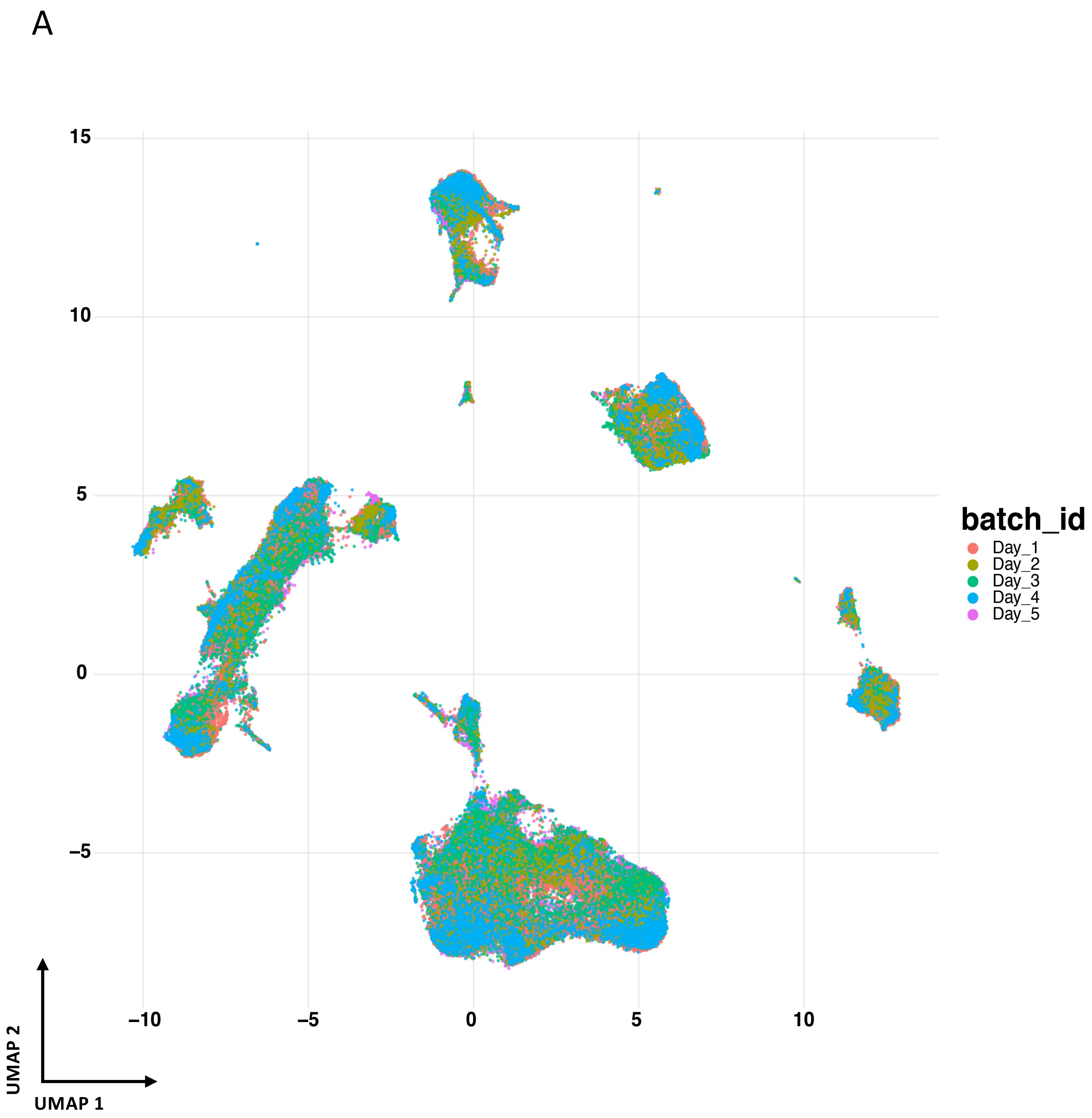

Supplementary Figure 1A.
UMAP graph overlaid for multiple batches (referred to different acquisition days).

B

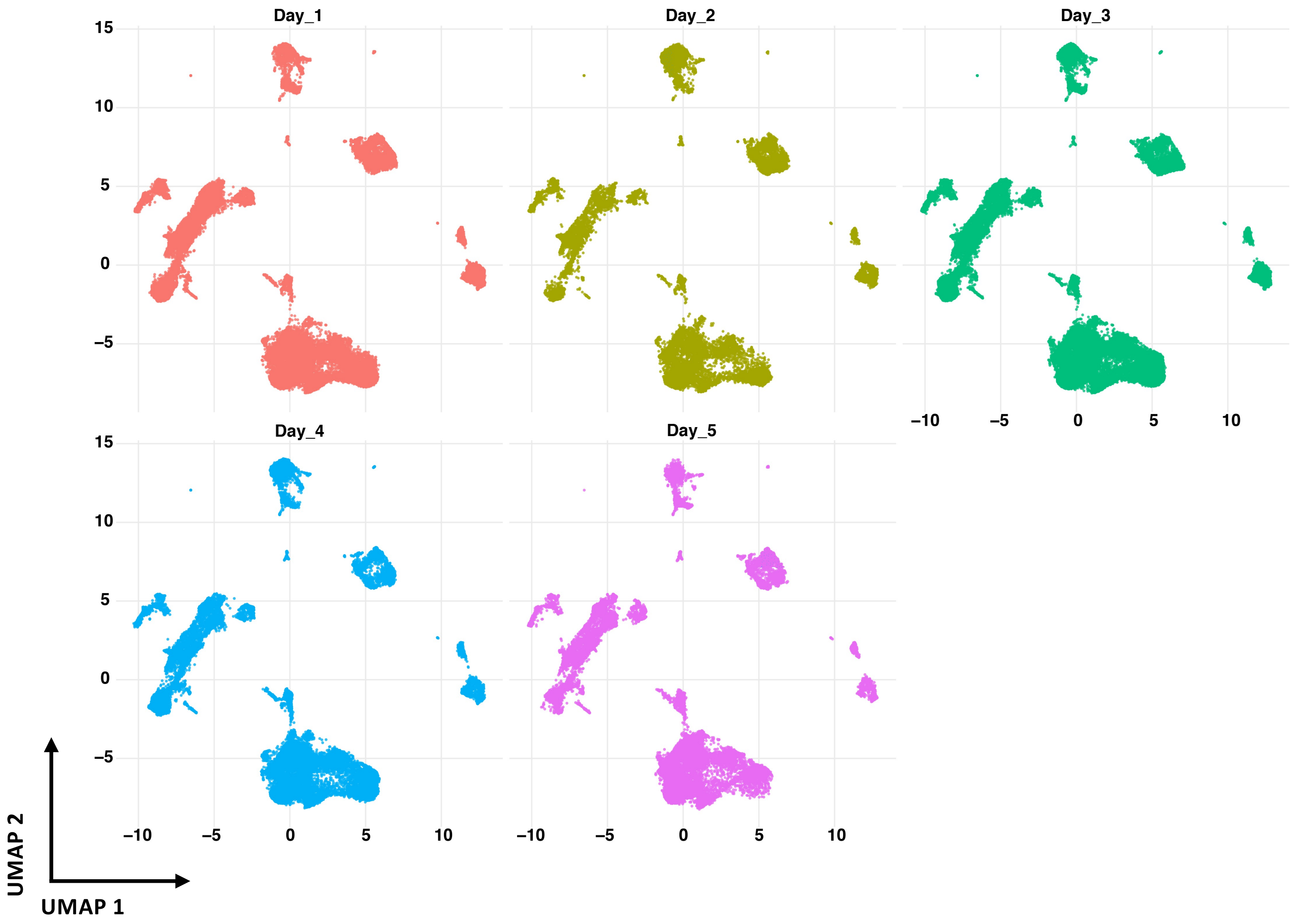

Supplementary Figure 1B.
Projection of UMAP graph stratified by batch; each color indicates a different day.

A

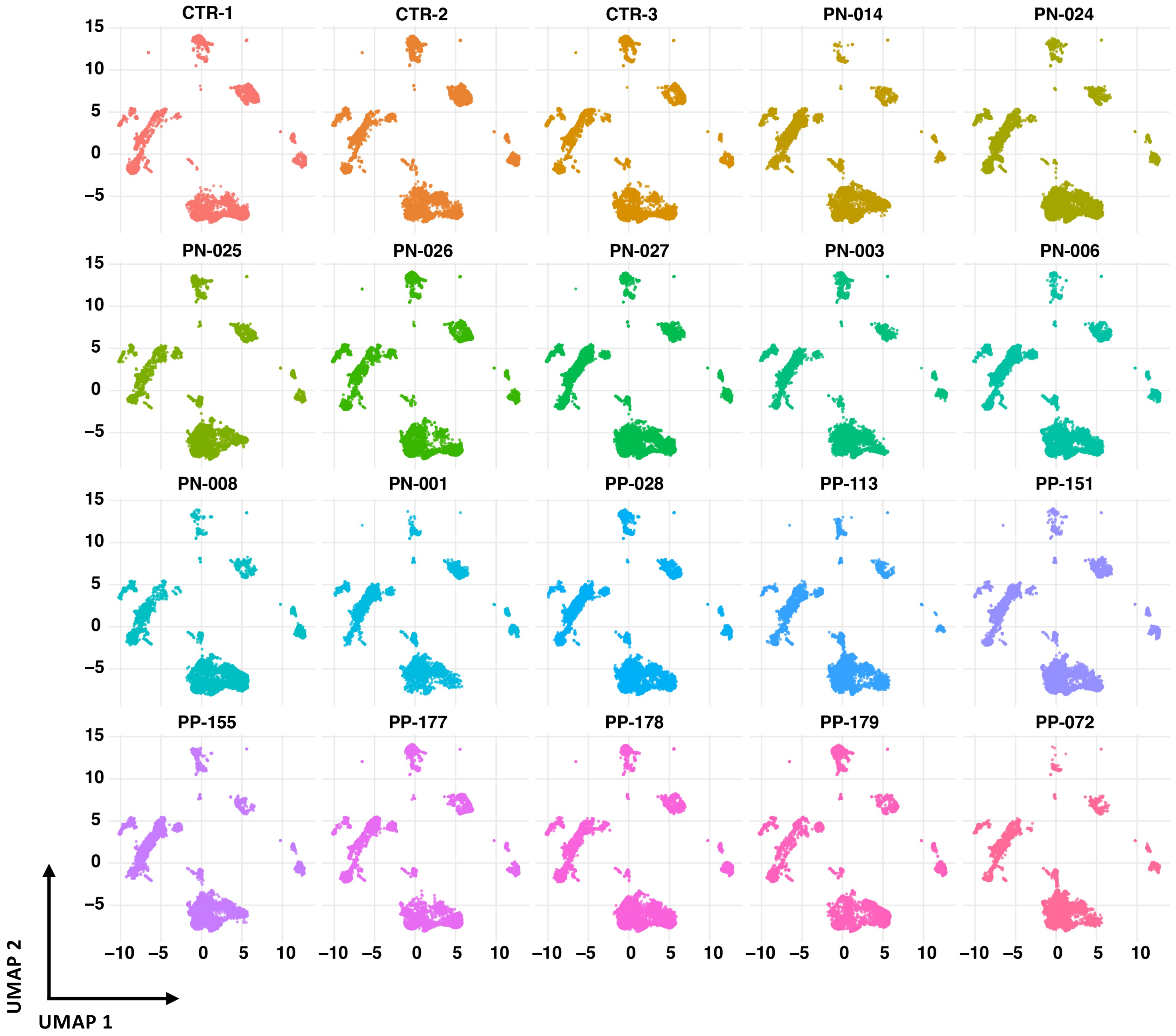

Supplementary Figure 2A.
UMAP graphs stratified by patient (CTR, healthy controls; PN, pregnant negative; PP, pregnant positive).

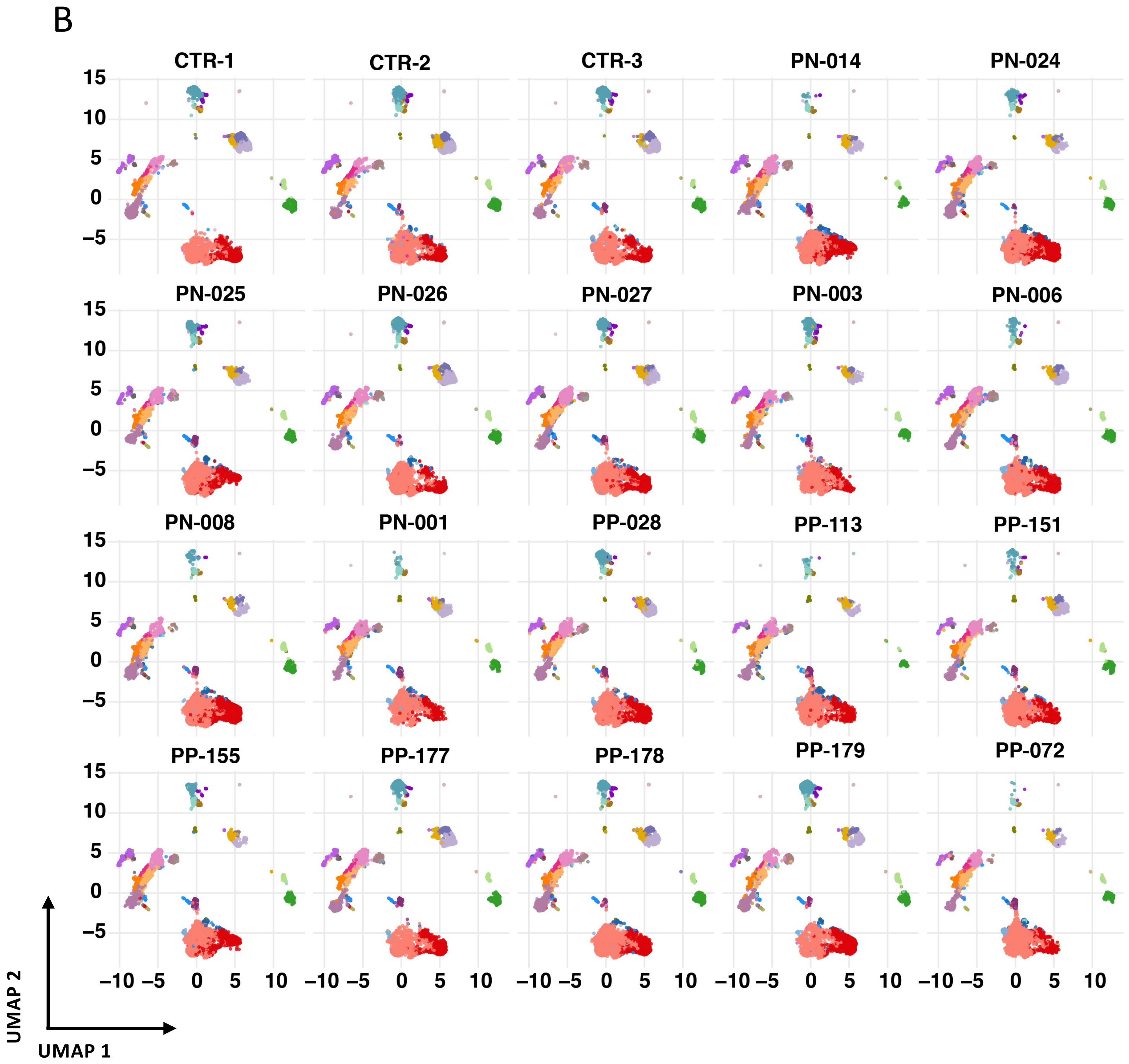

Supplementary Figure 2B.
Projection of UMAP graphs stratified by patient showing the FlowSOM clusters (CTR, healthy controls; PN, pregnant negative; PP, pregnant positive).

A

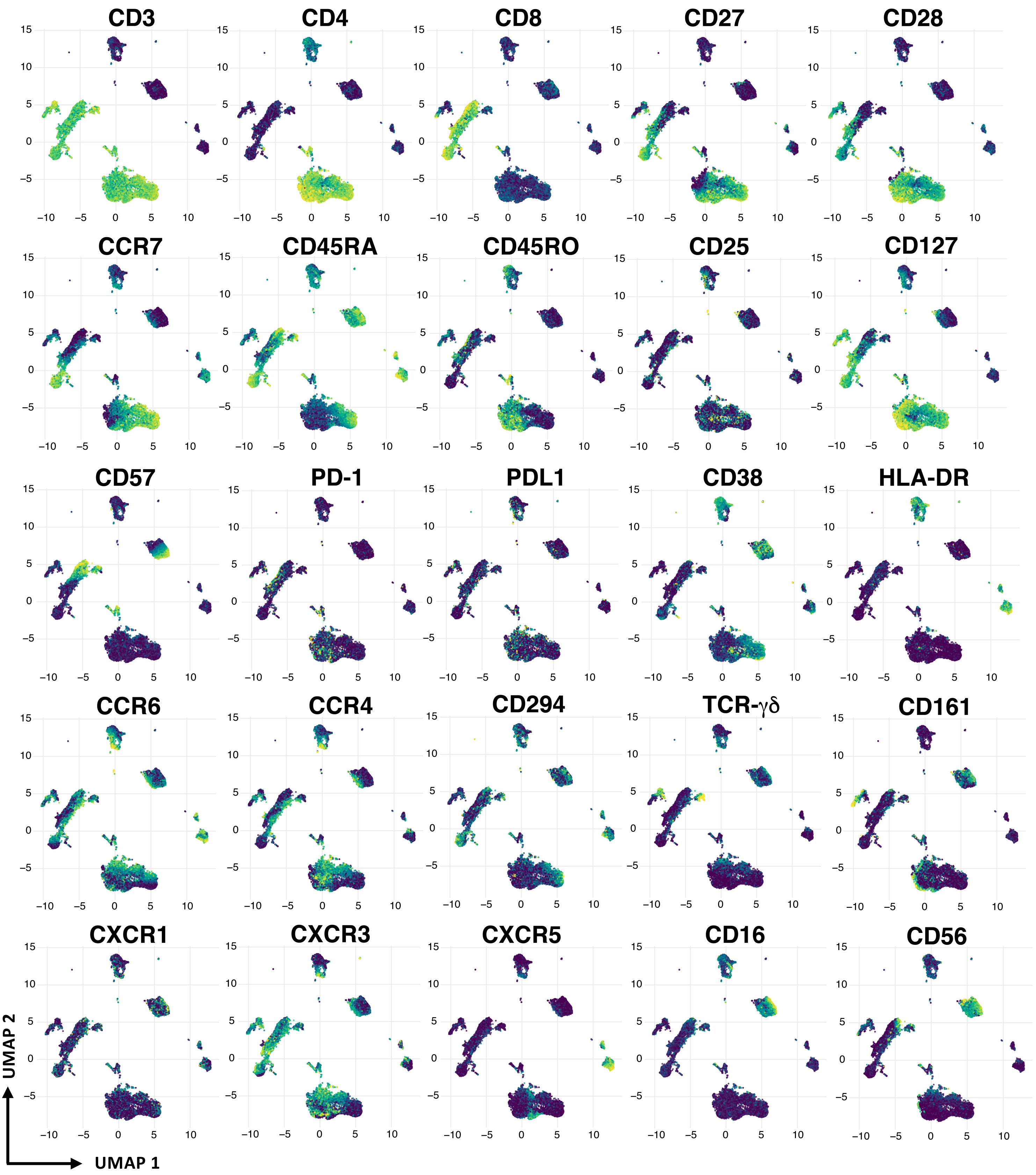

Supplementary Figure 3A.
UMAP graphs colored by the expression of 38 markers used for PBMC phenotyping.

B

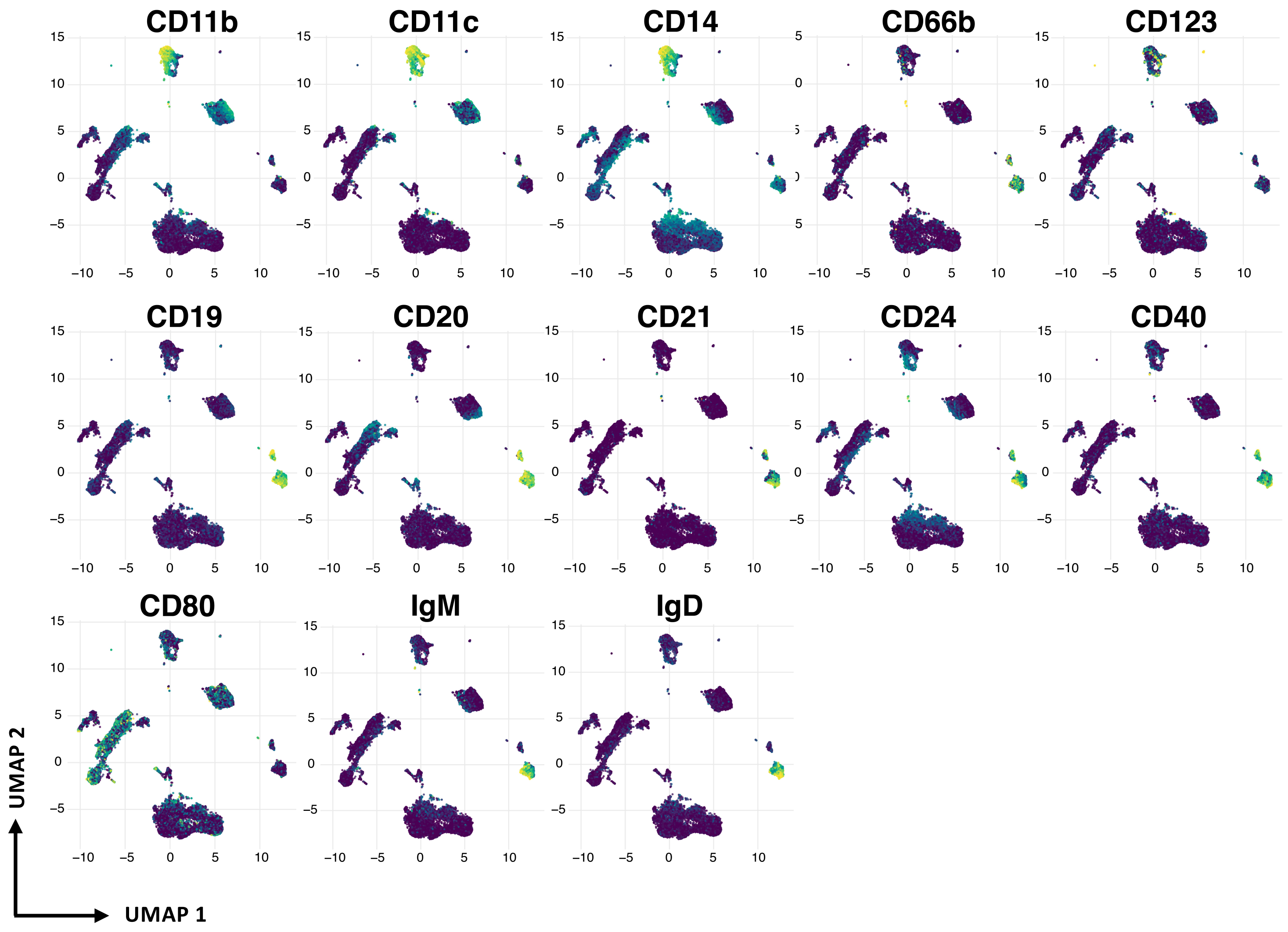

Supplementary Figure 3B.
UMAP graphs colored by the expression of 38 markers used for PBMC phenotyping.

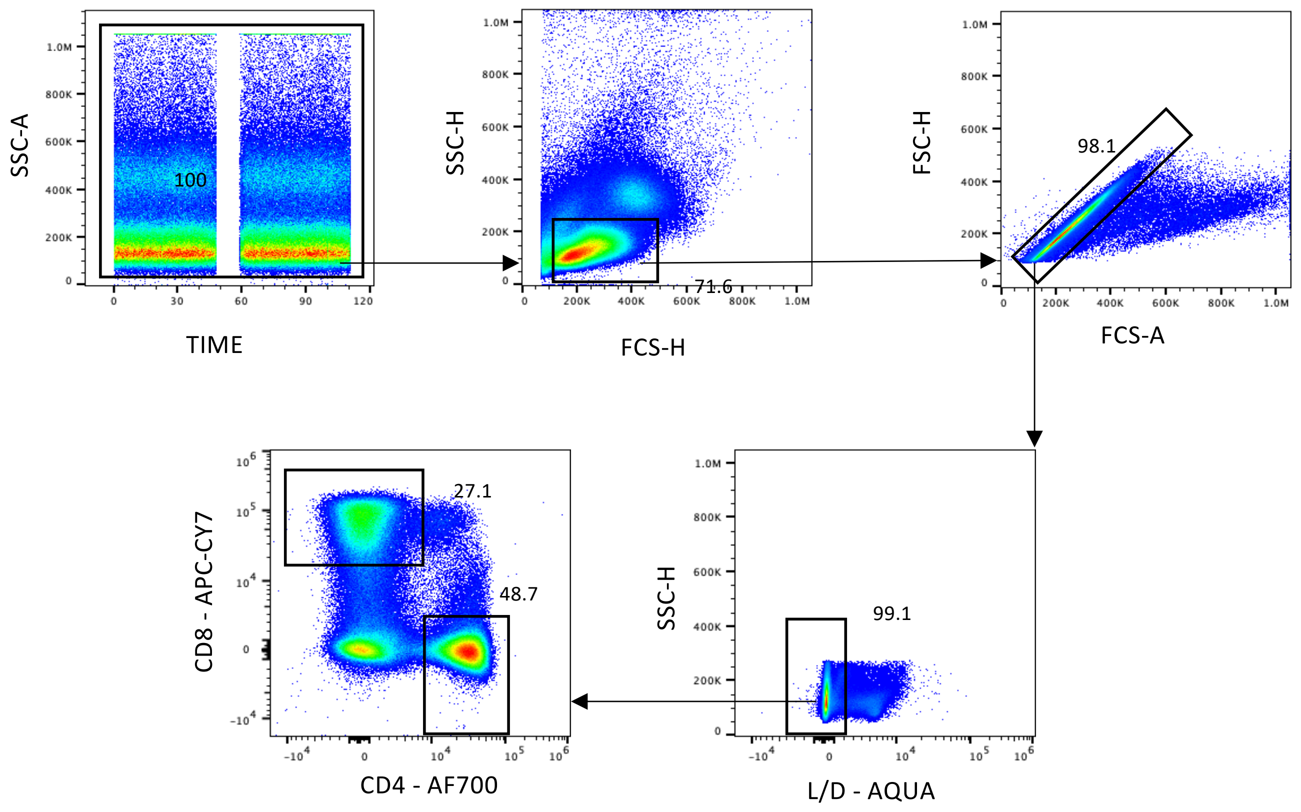

Supplementary Figure 4. Analysis of master regulator genes and chemokine receptors.
The figure shows the gating strategy used for detecting the expression of TH1-TH2-TH17 master regulator genes and chemokine receptors on $CD4^+$ and CD8 T^+ cells. Fluidic perturbancies were excluded from the analysis by the SSC-A vs Time gate, then lymphocyte were selected according to physical parameters. Doubles were removed according to FSC-A vs FSC-H gate and in this population live lymphocyte were selected. $CD4^+$ and $CD8^+$ T cells were identified according to the positivity of these markers. Boundaries between positive and negative were selected according to single stained tube and fluorescent minus one controls (FMO).

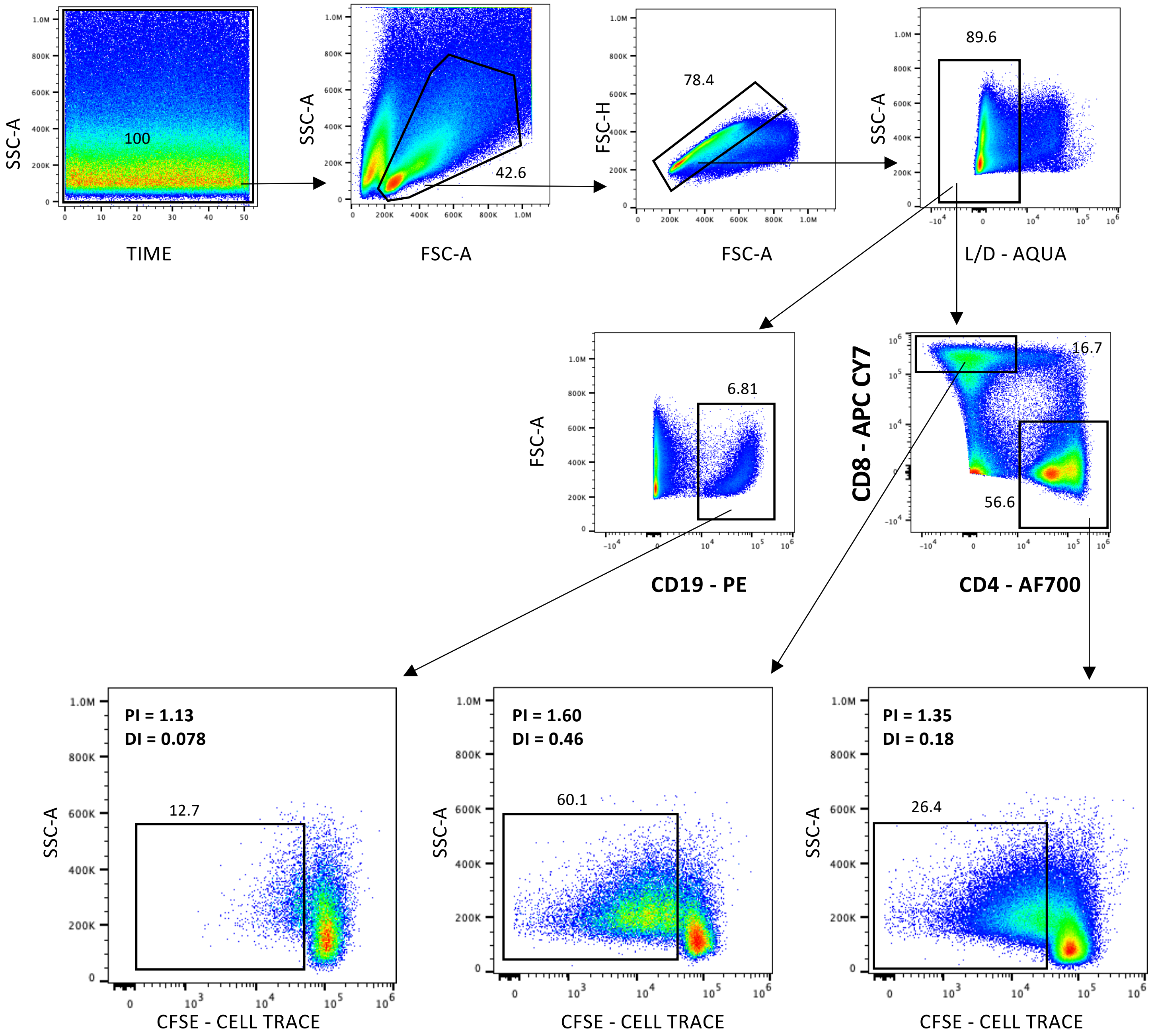

Supplementary Figure 5. Single cell analysis of T and B cell proliferative capability.
The figure shows the gating strategy used for the analysis of cell proliferation. Fluidic perturbancies were excluded from the analysis by the SSC-A vs Time gate, then lymphocyte were selected according to physical parameters. Doubles were removed according to FSC-A vs FSC-H gate. Among living cells (AQUA-negative), we identified B lymphocytes ($CD19^+$), helper ($CD4^+$) and cytotoxic ($CD8^+$) T cells. Then, we detected the proliferative capability of each cell population.

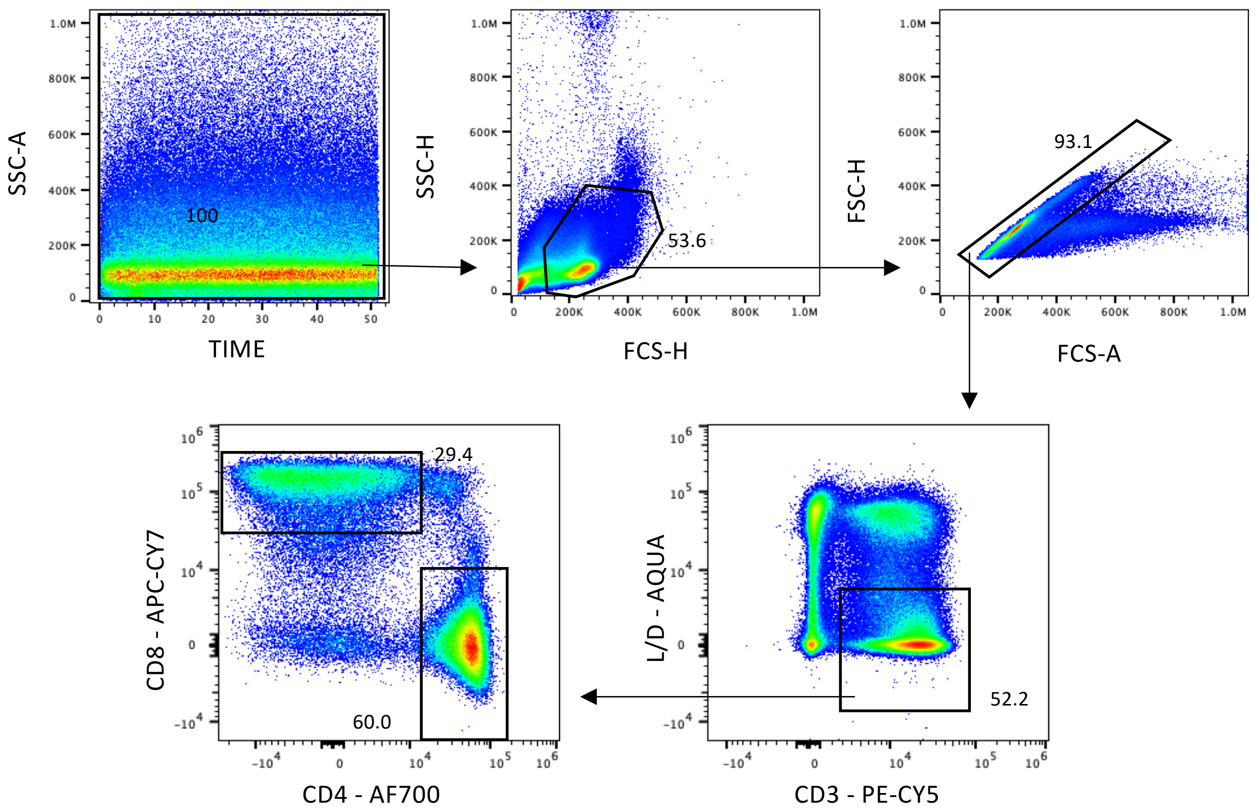

Supplementary Figure 6. Representative example of cytokine production by CD4$^+$ and CD8$^+$ T cells.
The figure shows the gating strategy used for the identification of CD4$^+$ and CD8$^+$ T lymphocytes (selected among AQUA-negative, CD3$^+$ cells) that produce different types of cytokines.
Fluidic perturbancies were excluded from the analysis by the SSC-A vs Time gate, then lymphocyte were selected according to physical parameters. Doubles were removed according to FSC-A vs FSC-H gate. Living CD3$^+$ T lymphocytes were selected and in this population CD4$^+$ and CD8$^+$ T lymphocytes were identified.

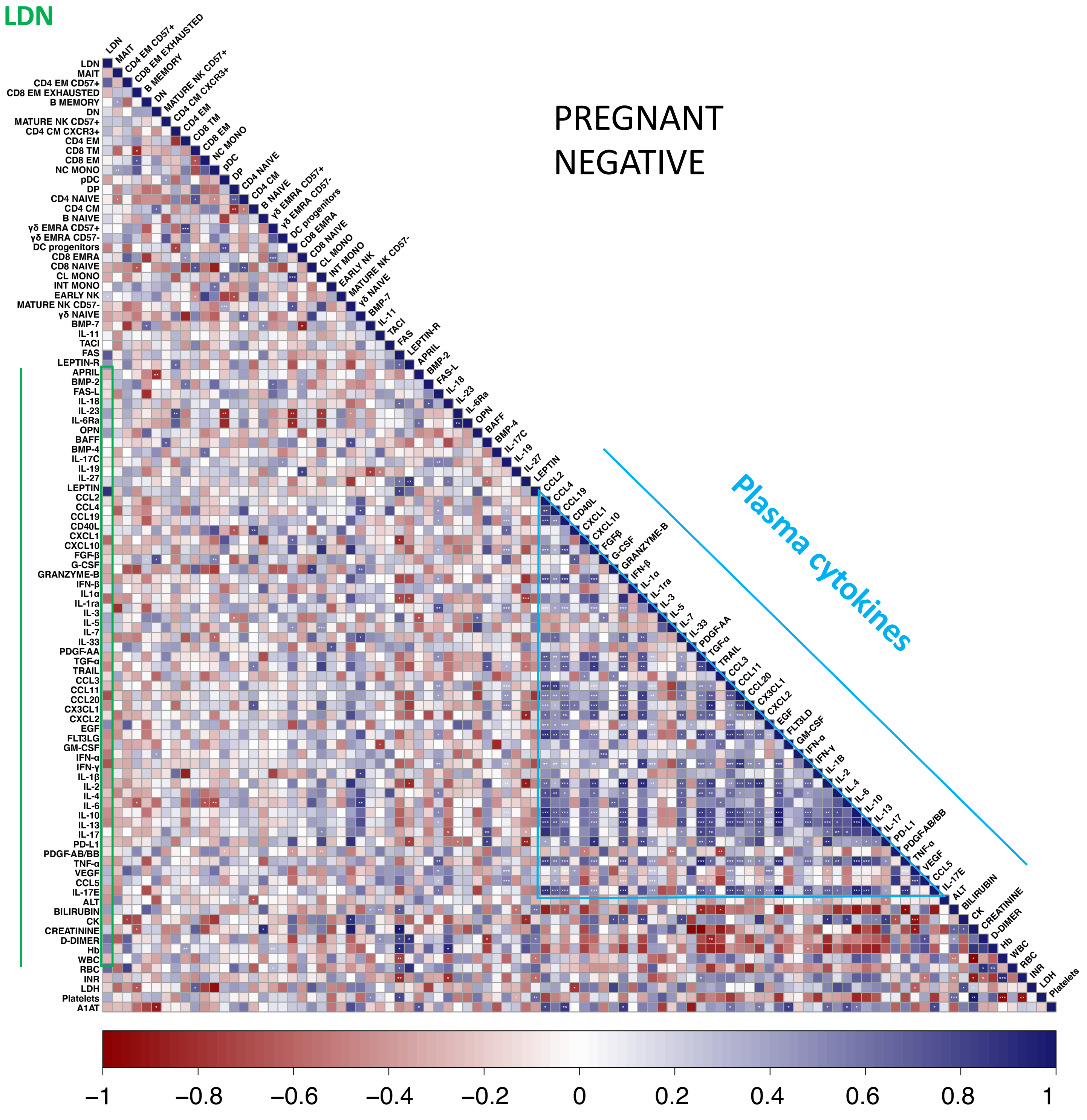

Supplementary Figure 7. Correlogram of pregnant negative women (PN). Spearman R values are shown from red (−1.0) to blue (1.0); r values are indicated by color. Additional information on feature names are described in the STAR Methods. Blank fields with dots indicate lack of signal. Spearman rank two-tailed p-value was indicated by *P < 0.05, **P < 0.01, and ***P < 0.001.
