# Supplementary material for: Endogenous control of inflammation characterizes pregnant women with asymptomatic or paucisymptomatic SARS-CoV-2 infection
Source: Nat Commun. 2021 Jul 29;12:4677. doi: 10.1038/s41467-021-24940-w (PMC8322155; doi:10.1038/s41467-021-24940-w)
Supplement: Supplementary file 3 — Reporting Summary [file 41467_2021_24940_MOESM3_ESM.pdf]

nature research

Corresponding author(s): ANDREA COSSARIZZA

Last updated by author(s): Jul 9, 2021

Reporting Summary

Nature Research wishes to improve the reproducibility of the work that we publish. This form provides structure for consistency and transparency in reporting. For further information on Nature Research policies, see our Editorial Policies and the Editorial Policy Checklist.

Statistics

For all statistical analyses, confirm that the following items are present in the figure legend, table legend, main text, or Methods section.

n/a	Confirmed	
☐	☒	The exact sample size (n) for each experimental group/condition, given as a discrete number and unit of measurement
☐	☒	A statement on whether measurements were taken from distinct samples or whether the same sample was measured repeatedly
☐	☒	The statistical test(s) used AND whether they are one- or two-sided<br>*Only common tests should be described solely by name; describe more complex techniques in the Methods section.*
☒	☐	A description of all covariates tested
☐	☒	A description of any assumptions or corrections, such as tests of normality and adjustment for multiple comparisons
☐	☒	A full description of the statistical parameters including central tendency (e.g. means) or other basic estimates (e.g. regression coefficient) AND variation (e.g. standard deviation) or associated estimates of uncertainty (e.g. confidence intervals)
☐	☒	For null hypothesis testing, the test statistic (e.g. F, t, r) with confidence intervals, effect sizes, degrees of freedom and P value noted<br>*Give P values as exact values whenever suitable.*
☒	☐	For Bayesian analysis, information on the choice of priors and Markov chain Monte Carlo settings
☒	☐	For hierarchical and complex designs, identification of the appropriate level for tests and full reporting of outcomes
☒	☐	Estimates of effect sizes (e.g. Cohen's d, Pearson's r), indicating how they were calculated

Our web collection on statistics for biologists contains articles on many of the points above.

Software and code

Policy information about availability of computer code

Data collection	Data in FCS file format were normalized for intra-file and inter-file signal drift using the FCS Processing tab in the CyTOF Software 6.7.<br>PBMC were acquired by using Attune NxT acoustic focusing flow cytometer (ThermoFisher). FCS data were acquired in list mode by Attune nxT Software v4.2.
Data analysis	Compensated and normalized Flow Cytometry Standard (FCS) 3.0 files were imported into FlowJo software version 10.<br>The main cell population identification was performed through unsupervised clustering using the FlowSOM v1.22.0<br>algorithm (K= 30). 2D visual representation was performed applying Uniform Manifold Approximation and Projection (UMAP). Statistical analysis was performed using generalized linear mixed models (GLMM) applying as FDR cutoff = 0.05.<br>All living CD45+ were exported for further analysis in R using Bioconductor libraries CATALYST (version 1.12.2) 21 and diffcyt (version 1.8.8).<br>Pairwise correlations between variables were calculated and visualized as a correlogram using R function corrplot v0.84.<br>Quantitative variables were compared using Mann-Whitney test. Statistical analyses were performed using Prism 6.0

For manuscripts utilizing custom algorithms or software that are central to the research but not yet described in published literature, software must be made available to editors and reviewers. We strongly encourage code deposition in a community repository (e.g. GitHub). See the Nature Research guidelines for submitting code & software for further information.

Data

Policy information about availability of data

All manuscripts must include a data availability statement. This statement should provide the following information, where applicable:

- Accession codes, unique identifiers, or web links for publicly available datasets
- A list of figures that have associated raw data
- A description of any restrictions on data availability

The flow cytometry data generated in this study have been deposited in the flowrepository.org database under accession code FR-FCM-Z3GH [https://flowrepository.org/experiments/3601]. The raw data generated in this study are provided in the Source Data file.

Field-specific reporting

Please select the one below that is the best fit for your research. If you are not sure, read the appropriate sections before making your selection.

☒ Life sciences ☐ Behavioural & social sciences ☐ Ecological, evolutionary & environmental sciences

For a reference copy of the document with all sections, see nature.com/documents/nr-reporting-summary-flat.pdf

Life sciences study design

All studies must disclose on these points even when the disclosure is negative.

Sample size	We have collected all pregnant women with SARS-CoV-2 infection who came consecutively to our attention in the period of the study, and gave informed consent, and those healthy pregnant women who agree to participated to the study.
Data exclusions	No data were excluded from the analysis, since all were technically correct.
Replication	The biological material that we obtained from the studied subjects was unique in the sense they after blood collection SARS-CoV-2 positive pregnant women went on quarantine, and those negative left the hospital, typically until delivery. Samples were collected from patients and controls, as allowed by the ethical commettee. For this reason, we could not replicate the experiments in different days. However, all measures were performed in technical duplicates (ELISA) or were performed on several millions cells (flow cytometry).
Randomization	We studied women who were negative or positive to SARS-CoV-2 infection, and we did not use any drug in them. So, there was no need of randomization.
Blinding	We had to told immediately women if they were negative or positive to SARS-CoV-2 infection. So, there is no blinding.

Reporting for specific materials, systems and methods

We require information from authors about some types of materials, experimental systems and methods used in many studies. Here, indicate whether each material, system or method listed is relevant to your study. If you are not sure if a list item applies to your research, read the appropriate section before selecting a response.

Materials & experimental systems

n/a	Involved in the study	
☐	☒	Antibodies
☒	☐	Eukaryotic cell lines
☒	☐	Palaeontology and archaeology
☒	☐	Animals and other organisms
☐	☒	Human research participants
☒	☐	Clinical data
☒	☐	Dual use research of concern

Methods

n/a	Involved in the study	
☒	☐	ChIP-seq
☐	☒	Flow cytometry
☒	☐	MRI-based neuroimaging

Antibodies

Antibodies used

Antibodies used for Cytof panel (indicated as target, clone, tag, note):
CD45, HI30, 89Y, MD-IPA
CD196/CCR6, G034E3, 141Pr, MD-IPA
CD123, 6H6, 143Nd, MD-IPA
CD19, HIB19, 144Nd, MD-IPA
CD4, RPA-T4, 145Nd, MD-IPA

CD8a, RPA-T8, 146Nd, MD-IPA
CD11c, Bu15, 147Sm, MD-IPA
CD16, 3G8, 148Nd, MD-IPA
CD45RO, UCHL1, 149Sm, MD-IPA
CD45RA, HI100, 150Nd, MD-IPA
CD161, HP-3G10, 151Eu, MD-IPA
CD194/CCR4, L291H4, 152Sm, MD-IPA
CD25, BC96, 153Eu, MD-IPA
CD27, O323, 154Sm, MD-IPA
CD57, HCD57, 155Gd, MD-IPA
CD183/CXCR3, G025H7, 156Gd, MD-IPA
CD185/CXCR5, J252D4, 158Gd, MD-IPA
CD28, CD28.2, 160Gd, MD-IPA
CD38, HB-7, 161Dy, MD-IPA
CD56/NCAM, NCAM16.2, 163Dy, MD-IPA
TCRgd, B1, 164Dy, MD-IPA
CD294, BM16, 166Er, MD-IPA
CD197/CCR7, G043H7, 167Er, MD-IPA
CD14, 63D3, 168Er, MD-IPA
CD3, UCHT1, 170Er, MD-IPA
CD20, 2H7, 171Yb, MD-IPA
CD66b, G10F5, 172Yb, MD-IPA
HLA-DR, LN3, 173Yb, MD-IPA
IgD, IA6-2, 174Yb, MD-IPA
CD127, A019D5, 176Yb, MD-IPA
Cell-ID Intercalator-103Rh, -, 103Rh, MD-IPA
CD181/CXCR1, 8F1/CXCR1, 142Nd, 3142009B
CD274/PDL1, 29E.2A3, 159Tb, 3159029B
CD80/B7.1, 2D10.4, 162Dy, 3162010B
CD40, 5C3, 165Ho, 3165005B
CD24/PD-1, EH12.2H7, 175Lu, 3175008B
CD11b/Mac-1, ICRF44, 209Bi, 3209003B
CD21, NA, 116Cd, Custom
IgM, NA, 114Cd, Custom

Antibodies used for the identification of master regulator genes, chemokine receptors and intracellular cytokine staining (indicated as target, dye, clone, vendor, cat number, lot number):
LIVE DEAD, AQUA, NA, thermoFisher, L34965, 2157151
CXCR3, AF488, 1C6/CXCR3, BECTON DICKINSON, 558047, 374115
CXCR4, PE, 12G5, BIOLEGEND, 306506, B296106
CD161, PC7, 191B8, BECKMAN COULTER, B30631, 200022
CCR6, BV605, G034E3, BIOLEGEND, 353420, B291940
CCR4, PE-CF594, 1G1, BECTON DICKINSON, 565391, 7276847
CD4, AF700, RPA-T4 BIOLEGEND, 300526, B313478
CD8, APC-CY7, RPA-T8, BIOLEGEND, 301016, B274260
GATA3, BV421, 16E10A23, BIOLEGEND, 653814, B301731
TBET, APC, 4B10, BIOLEGEND, 644814, B296978
CD19, PE, HIB19, BIOLEGEND, 302208, B273506
CFSE, AF488, NA, THERMOFISHER, C34554, 1255942
CD3, PE-CY5, UCHT1, BIOLEGEND, 300410, B270168
IL-4, APC, MMQ1-17H12, BIOLEGEND, 500310, B294390
IL-17, BV421, BL168, BIOLGENED, 512322, B192196
TNF, BV605, MAB11, BIOLEGEND, 502936, B312493
IFN-G, FITC, B27, BIOLEGEND, 506504, B286029
CD107A, PE, H4A3, BIOLEGEND,328608

Validation

In the last 20 years or more, all the antibodies have been validated for flow cytometry use in the field of human research by Becton Dickinson, Biolegend, Beckman Coulter and Fluidigm. There is a huge number of papers in the literature that have described, characterized and used such reagents, whose use is of routine in most labs.

Human research participants

Policy information about studies involving human research participants

Population characteristics	A total of 14 pregnant women with SARS-CoV2 infection was included in the study; they had a median age of 33.8 years (range 19-39). Patients were matched for age and gender with 28 pregnant women negative to nasopharyngeal swab (median 33.9 years, range 18-42) and a total of 15 non pregnant healthy women (CTR), median age 39 years (range 25-50 years). We recorded demographic data, medical history, symptoms, signs, temperature, and main laboratory findings from each patient. For details, see supplementary Table 1.
Recruitment	We enrolled 14 SARS-CoV-2 positive and 28 SARS-CoV-2 negative pregnant women who came consecutively to our clinics for routine visits, and accepted to participate to the study by donating their blood. There was no bias in the patient selection.
Ethics oversight	This is a case-control, cross sectional, single-centre study, approved by the local Ethical Committee (Comitato Etico dell'Area Vasta Emilia Nord, protocol number 177/2020, March 11th, 2020) and by the University Hospital Committee (Direzione Sanitaria dell'Azienda Ospedaliero-Universitaria di Modena, protocol number 7531, March 11th, 2020).

Note that full information on the approval of the study protocol must also be provided in the manuscript.

Flow Cytometry

Plots

Confirm that:

- [x] The axis labels state the marker and fluorochrome used (e.g. CD4-FITC).
- [x] The axis scales are clearly visible. Include numbers along axes only for bottom left plot of group (a 'group' is an analysis of identical markers).
- [x] All plots are contour plots with outliers or pseudocolor plots.
- [x] A numerical value for number of cells or percentage (with statistics) is provided.

Methodology

Sample preparation	Blood samples (up to 20 mL) were obtained after informed consent. In some donors, blood was obtained after diagnosis of SARS-CoV-2 infection. Peripheral blood mononuclear cells (PBMC) were isolated according to standard procedures and stored in liquid nitrogen until use. Plasma was collected and stored at -80°C until use. Measurements were taken from individual patients; in the case of plasma, each measurement was performed in duplicate and only the mean was considered and shown.
Instrument	Attune NxT acoustic flow Cytometer (ThermoFisher), CyTOF Helios (Fluidigm).
Software	FlowJo X, Prism 6.0, Attune NxT software, CyTOF Software 6.7
Cell population abundance	We have investigated all cells obtained from the blood collection, that was performed respecting the indications given by the local ethical committee that approved the study.
Gating strategy	Fluidic perturbancies were excluded from the analysis by the SSC-A vs Time gate, then lymphocyte were selected according to physical parameters. Doubles were removed according to FSC-A vs FSC-H gate and in this population live lymphocyte were selected. CD4 and CD8 T cells were identified according to the positivity to mAbs recognizing these markers. Boundaries between positive and negative were selected according to single stained tubes and "fluorescent minus one controls" (FMO), according to the state-of-the-art cytometry.

- [x] Tick this box to confirm that a figure exemplifying the gating strategy is provided in the Supplementary Information.
