# Supplementary material for: Endogenous control of inflammation characterizes pregnant women with asymptomatic or paucisymptomatic SARS-CoV-2 infection
Source: Nat Commun. 2021 Jul 29;12:4677. doi: 10.1038/s41467-021-24940-w (PMC8322155; doi:10.1038/s41467-021-24940-w)
Supplement: Supplementary file 4 — Description of Additional Supplementary Files [file 41467_2021_24940_MOESM4_ESM.pdf]

Description of Additional Supplementary Files

File Name: Supplementary Data 1
Description: Clinical and hematological data in pregnant women. Fourteen women with paucisymptomatic or asymptomatic SARS-CoV-2 infection are compared to 28 healthy pregnant women. Exact p value, obtained by two-sided Mann Withney test, is indicated in the bottom line.

File Name: Supplementary Data 2
Description: The table shows the percentage of different clusters of peripheral blood mononuclear cells, as obtained by unsupervised analysis of samples from pregnant womed negative or positive to SARS-CoV-2 infection (in blue). The table also reports the plasmatic level of the cytokines that were measured (orange), along with the laboratory parameters (violet) that were used to perform the correlation analysis of pregnant women positive and negative for SARS-CoV-2.

File Name: Supplementary Data 3
Description: The matrix, related to the data obtained in SARS-CoV-2 negative pregnant women (i.e., different proportions of PBMC populations, plasma cytokine levels, laboratory parameters), reports all Spearman rank two-tailed exact p-value calculated using cor.mtest function of corrplot v0.84.

File Name: Supplementary Data 4
Description: The matrix, related to the data obtained in SARS-CoV-2 positive pregnant women (i.e., different proportions of PBMC populations, plasma cytokine levels, laboratory parameters), reports all Spearman rank two-tailed exact p-value calculated using cor.mtest function of corrplot v0.84.

File Name: Supplementary Data 5
Description: List of lyophilized antibodies used in mass cytometry assays. Columns indicate the recognized target molecule, the name of the monoclonal antibody clone, the tag (i.e., the isotope used for any clone) and the code of the product.

File Name: Supplementary Data 6
Description: List of antibodies used in flow cytometry assays for the identification of master regulator genes, chemokine receptors and intracellular cytokine staining. Columns indicate the recognized target molecule, the fluorochrome used for any clone, the name of the vendor, the catalogue number, the lot number and the titer at which the reagent was used.
